# Successful resolution of gastric pneumatosis due to a strangulated hiatus hernia and malrotation through non-surgical management: a case report

**DOI:** 10.1093/bjrcr/uaaf007

**Published:** 2025-02-05

**Authors:** Shiv Hadani, Dhaara Bhatt, Ashish Bhagat, Vivek Malhotra

**Affiliations:** Radiology Department, West Hertfordshire Teaching Hospitals NHS Trust, Watford General Hospital, Watford, Hertfordshire, WD18 0HB, United Kingdom; Radiology Department, West Hertfordshire Teaching Hospitals NHS Trust, Watford General Hospital, Watford, Hertfordshire, WD18 0HB, United Kingdom; Radiology Department, West Hertfordshire Teaching Hospitals NHS Trust, Watford General Hospital, Watford, Hertfordshire, WD18 0HB, United Kingdom; Radiology Department, West Hertfordshire Teaching Hospitals NHS Trust, Watford General Hospital, Watford, Hertfordshire, WD18 0HB, United Kingdom

**Keywords:** gastric pneumatosis, hiatus hernia, malrotation

## Abstract

Gastric pneumatosis is a rare finding, and clinicians, when under pressure, find it challenging to immediately identify the cause and decide if the patient requires emergency surgery. We present a case where an initial CT scan demonstrated gastric pneumatosis involving only the greater curvature of the antrum caused by a strangulated hiatus hernia and malrotation of the distal stomach. The CT features suggested the patient required immediate surgery; however, a conservative approach was taken, and a follow-up CT scan 4 days after the onset demonstrated complete resolution and no long-term complications.

## Case history

A 53-year-old female presented to the emergency department with a sudden onset of abdominal pain and coffee-ground vomiting. Her past medical history included cerebral palsy, epilepsy, hypertension, and anaemia. The patient denied taking any non-steroidal anti-inflammatory drugs (NSAIDs) along with her regular medications for her long-term conditions. On examination, the patient had generalized abdominal tenderness and guarding, bowel sounds were present on auscultation, and there were no features of peritonism. Other systems were unremarkable.

**Figure 1.**
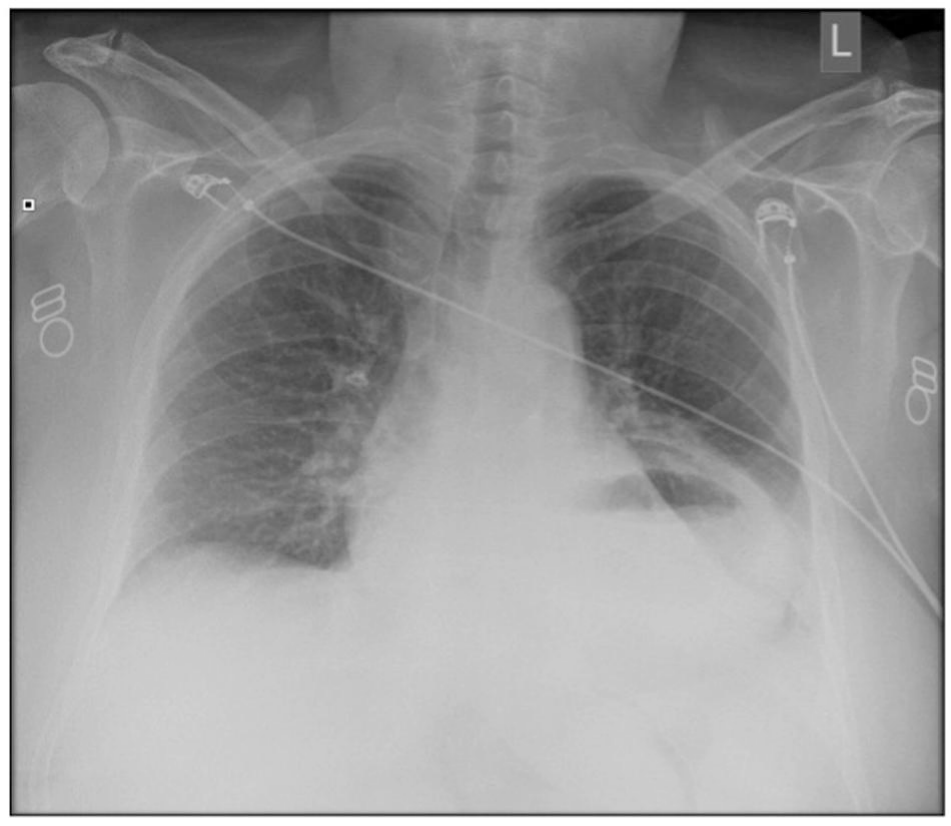
Initial radiograph demonstrating fluid-filled cavity in the chest.

Basic observations demonstrated an elevated blood pressure and heart rate and an electrocardiogram (ECG) with normal sinus rhythm. Laboratory blood tests demonstrated an elevated white cell count, c-reactive protein (CRP) and amylase, and blood gas showed a normal pH of 7.41 and a normal lactate of 2.10. A chest radiograph revealed a prominent air-fluid level in the chest in keeping with a hiatus hernia (Figure 1).

The patient was kept nil by mouth, analgesia, intravenous (IV) fluids, and proton pump inhibitors (PPIs) were prescribed, and their regular medications were changed from the oral route. The medical team requested a CT scan of the abdomen and pelvis to rule out perforation, given the presenting features.

**Figure 2.**
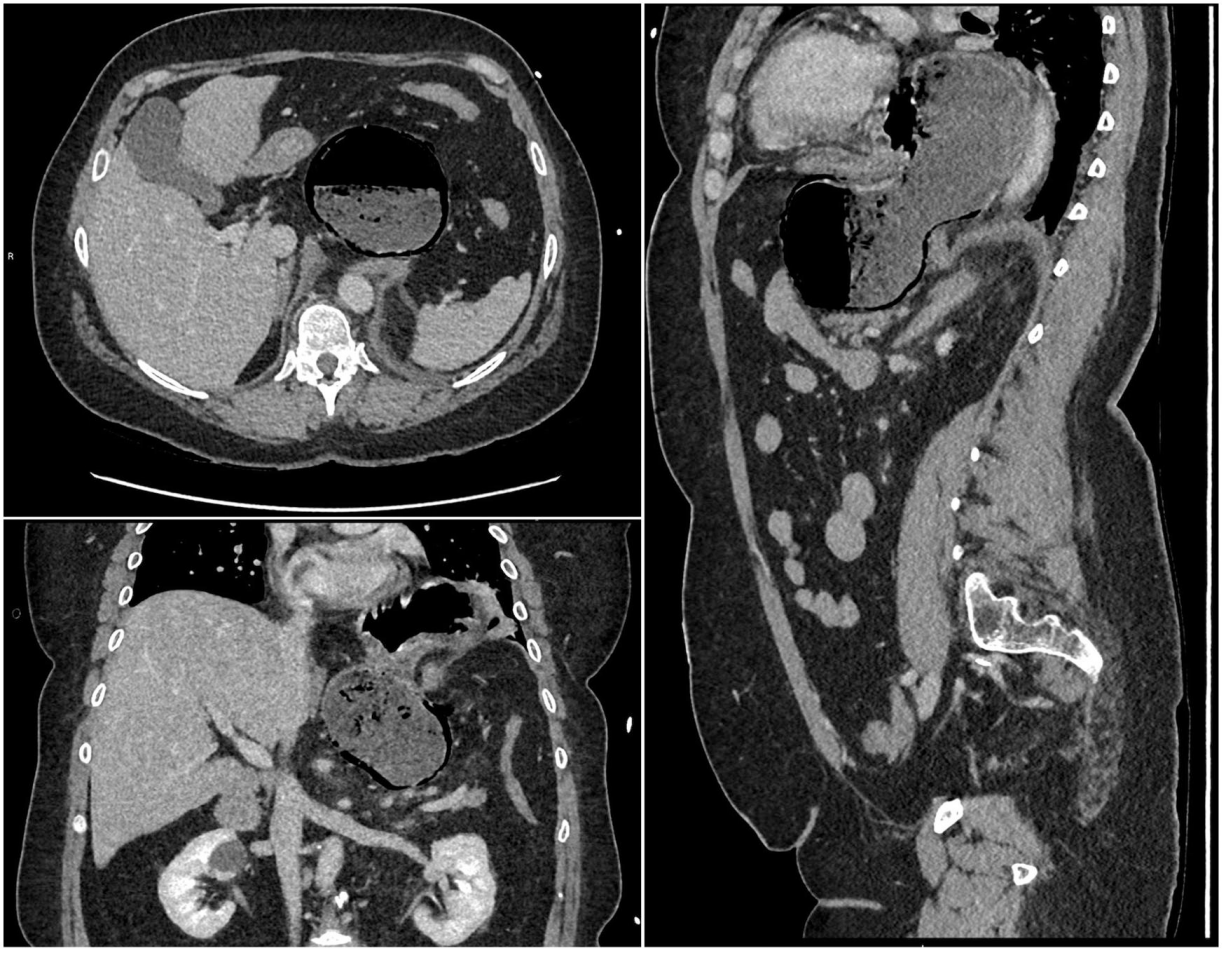
Axial, coronal, and sagittal view of gastric pneumatosis, strangulated hiatus hernia, and malrotation of the stomach at initial presentation.

The initial CT scan was done in the portal venous phase. The CT demonstrated herniation and distention of the body and fundus of the stomach into the left chest cavity, clockwise malrotation of the distal stomach, and gastric pneumatosis involving only the greater curvature of the antrum (Figure 2). There was no portal venous gas, no occlusion of major blood vessels, and no dilated distal bowel loops. The CT scan was initially reported as a strangulated hiatus hernia with features concerning for gastric ischaemia.

**Figure 3.**
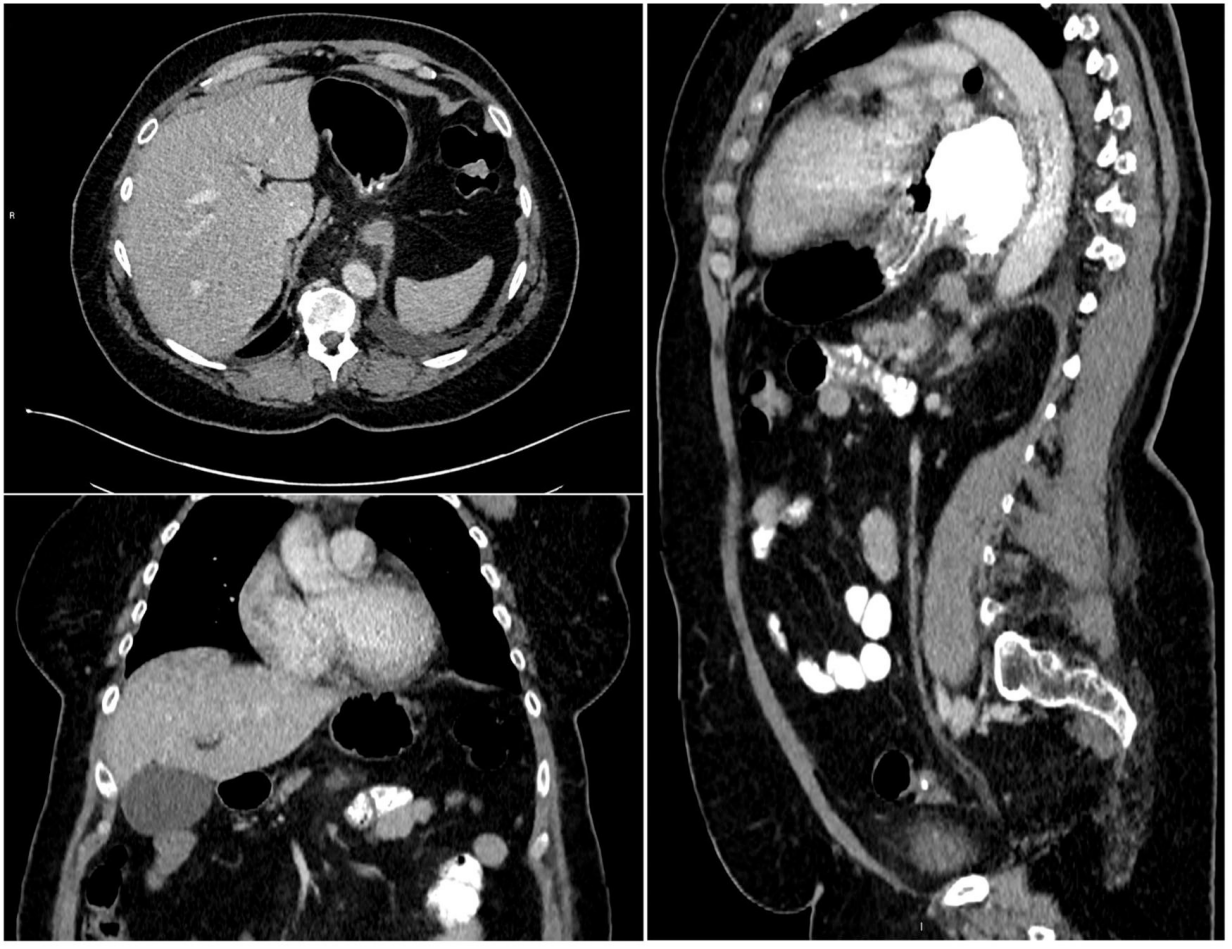
Axial, coronal, and sagittal view after 4 days of conservative management demonstrating resolution of gastric pneumatosis, the persistence of hiatus hernia, the passage of oral contrast into the small bowel and no perforation.

The duty radiologist immediately conveyed the findings to the medical team, and the patient was transferred to the surgical team. The surgical team sought further advice from the duty radiologist about whether a nasogastric (NG) tube could be successfully inserted to decompress the stomach. The duty radiologist advised attempting to insert the NG tube because there was no CT evidence of obstruction or tightening at the gastro-oesophageal junction (GOJ). The surgeons successfully inserted the NG tube and organized an urgent oesophagogastroduodenoscopy (OGD). The medical team performing the OGD found it difficult to navigate the scope into the stomach because of resistance at the lower oesophagus and GOJ, and the scope could not be passed beyond the pylorus. The stomach could also not be retroflexed by the scope due to the lack of space in the stomach to manoeuvre the scope. The medical team could not identify endoscopic evidence of gastric ischaemia, and hence, the surgical team decided to continue with conservative management. The surgical team also decided against performing a follow-up OGD to check for resolution because the patient was deemed at very high risk of developing complications. A follow-up CT scan with oral and IV contrast was instead performed 4 days after the onset. Oral contrast was used to confirm the passage of contrast from the stomach into the small bowel and rule out perforation. The follow-up CT demonstrated complete resolution of gastric pneumatosis, the stomach fundus placed back into the abdominal cavity, oral contrast passing from the stomach into the small bowel and no evidence of perforation (Figure 3). The patient was discharged and booked for an elective repair for their hiatus hernia.

## Discussion

Gastric intramural air, otherwise known as gastric pneumatosis, is reported to be a rare finding because the stomach has a rich collateral blood supply.^1–3^ The literature recognizes gastric emphysema and emphysematous gastritis as the 2 main differentials for gastric pneumatosis.^2^ Both differentials often confuse clinicians but have different causes.^2^

Gastric emphysema is the dissection of air into the stomach lining and disruption to the gastric mucosa integrity.^2^ Causes of gastric emphysema are non-infective and include gastric ischaemia, high intragastric pressure due to gastric outlet obstruction, iatrogenic due to instrumentation, severe vomiting, and dissection of air from the mediastinum into the stomach lining.^2^^,^^4^ Emphysematous gastritis is an infection of the stomach where the air is produced by gas-producing micro-organisms.^2^^,^^4^  *Escherichia coli*, *Streptococcus* species, *Enterobacter* species, and *Pseudomonas aeruginosa* are the most common micro-organisms causing emphysematous gastritis.^2^^,^^4^ Risk factors for developing emphysematous gastritis include diabetes mellitus, alcohol abuse, previous gastric surgery, gastroenteritis, immunosuppression, ingestion of corrosive materials, and taking medications regularly that disrupt the gastric mucosa, such as NSAIDs.^2^^,^^4^ Hence, identifying the causes and associated risk factors can initially assist the clinician in determining the likely differential.

The clinical presenting features of gastric emphysema and emphysematous gastritis overlap but vary in severity. Signs and symptoms in both entities include abdominal pain of varying severity, nausea, vomiting, haematemesis, and melaena.^1^^,^^2^^,^^4^ Patients with emphysematous gastritis also present with a fever.^1^^,^^2^^,^^4^ Patients with gastric emphysema are haemodynamically stable, and patients with emphysematous gastritis are haemodynamically unstable.^1^^,^^2^^,^^4^ If left untreated, gastric emphysema has a mortality of 29%, and emphysematous gastritis has a mortality nearly double that of 55%.^5^ Hence, clinicians need to confirm the correct differential early because of the overlap of symptoms and the high mortality associated with emphysematous gastritis compared to gastric emphysema.

CT is the imaging modality of choice to investigate patients presenting with an acute abdomen and is the most sensitive and specific imaging modality.^2^^,^^4^  Table 1 summarizes the CT features documented in the literature that help distinguish between gastric emphysema and emphysematous gastritis. However, the features are only suggestive as the patient may unknowingly convert from having gastric emphysema to developing emphysematous gastritis.^2^^,^^4^

**Table 1.** Gastric pneumatosis pattern and supporting features on CT for gastric emphysema and emphysematous gastritis.^2^^,^^4^

	Gastric emphysema	Emphysematous gastritis
Gastric pneumatosis pattern	Round air bubbles in the intramural wall	Streaky/mottled/linear air
Other supporting features	Presence or absence of portal venous gas	Presence or absence of portal venous gas Stomach wall thickening

The patient is initially managed conservatively in gastric emphysema and emphysematous gastritis.^3^^,^^6^ An NG tube is inserted, and PPIs, broad-spectrum antibiotics, and IV fluids for resuscitation are prescribed.^3^^,^^6^

An endoscopy is performed if there is either a clinical suspicion, to confirm and/or determine the severity of gastric ischaemia and to rule out other causes of gastric pneumatosis.^3^^,^^6^ Known endoscopic features of gastric emphysema include redness, erosion, coarse mucosa, ulceration and a clear boundary between normal healthy mucosa and abnormal gastric emphysematous mucosa.^7^ These features are commonly found in the longitudinal folds of the stomach on either the posterior wall, greater curvature or lesser curvature.^7^ However, distinguishing between gastric emphysema and emphysematous gastritis on endoscopy still requires further research.^7^

Surgery is generally indicated if there is a poor response and/or deterioration despite conservative measures and there is CT and/or endoscopic evidence of gastric ischaemia or perforation.^1^^,^^2^^,^^4^ The patient’s performance status is also considered before deciding whether to pursue surgical management.^1^^,^^2^^,^^4^

The authors speculated why only the greater curvature of the antrum showed gastric pneumatosis and the rest of the stomach appeared unremarkable. It is known that the right gastroepiploic artery and left gastroepiploic artery anastomose to supply the greater curvature of the stomach, and the right gastroepiploic artery predominantly supplies the antrum of the greater curvature.^8^ On reviewing the CT images, the authors speculated that clockwise malrotation of the distal portion of the stomach caused kinking and obstruction of the right gastroepiploic artery, and the hiatus hernia caused obstruction of the left gastroepiploic artery. Therefore, the overall supply of oxygenated nutrient-rich blood to the greater curvature of the antrum is reduced, causing gastric ischaemia and, hence gastric pneumatosis.

We present a unique case, where gastric pneumatosis involved only the greater curvature of the antrum. There are no reports in the literature to our knowledge of gastric pneumatosis involving only the greater curvature of the antrum. Previous case reports have demonstrated complete resolution of gastric pneumatosis through non-surgical management.^9^^,^^10^ Our case also demonstrated successful non-surgical management of gastric pneumatosis by inserting an NG tube and decompressing the stomach. The patient did not require life-changing emergency surgery and developed no complications following resolution. Hence, the patient’s hiatus hernia could be followed up and repaired on an elective basis.

## Conclusion

Gastric pneumatosis is a rare finding, and knowing whether this is due to gastric emphysema or emphysematous gastritis in an emergency setting is challenging. Obtaining a thorough clinical history, closely monitoring haemodynamic stability and imaging through CT helps to distinguish between the 2 entities and dictate management. Immediate surgery may not be required, and patients can be successfully managed through conservative measures. The case demonstrates the role of radiology in diagnosing and managing gastric pneumatosis, and the merit of using CT to monitor resolution in patients who may not be fit for invasive investigations and/or surgery. Further research is required to definitively differentiate between gastric emphysema and emphysematous gastritis on CT and the associated endoscopic findings of emphysematous gastritis.

## Learning points

- A thorough clinical history, close monitoring of haemodynamic stability and a focused CT scan, helps to distinguish between gastric emphysema and emphysematous gastritis as the cause of gastric pneumatosis.
- Radiologists should be familiar with the appearance of gastric pneumatosis on imaging and identify any supporting features and associated complications to aid the clinical team in distinguishing between gastric emphysema and emphysematous gastritis.
- Gastric pneumatosis when confirmed, should prompt an urgent surgical review to decide if immediate surgery is needed, although surgery may not be required, and patients can successfully be managed conservatively.
- A follow-up CT scan can be considered a safe investigation to monitor for resolution of gastric pneumatosis following conservative management in patients who are not fit for further invasive investigations and/or surgical intervention.

## Informed consent

Written, informed consent was obtained from the patient for publication of this case report, including the accompanying images.
